# MBD1 promotes the malignant behavior of gallbladder cancer cells and induces chemotherapeutic resistance to gemcitabine

**DOI:** 10.1186/s12935-019-0948-1

**Published:** 2019-09-09

**Authors:** Liu Wensheng, Zhang Bo, Hu Qiangsheng, Xu Wenyan, Ji Shunrong, Xu Jin, Ni Quanxing, Yu Xianjun, Xu Xiaowu

**Affiliations:** 1 Department of Pancreatic Surgery, Fudan University Shanghai Cancer Center, 200032 Shanghai, China; 2 Pancreatic Cancer Institute, Fudan University, 200032 Shanghai, People’s Republic of China; 3 Shanghai Pancreatic Cancer Institute, 200032 Shanghai, China

**Keywords:** Gallbladder cancer, Malignant behavior, Chemotherapeutic resistance, Methyl-CpG binding domain protein 1, Epithelial–mesenchymal transition

## Abstract

**Background:**

Methyl-CpG binding domain protein 1 (MBD1), which couples DNA methylation to transcriptional repression, has been implicated in transcriptional regulation, heterochromatin formation, genomic stability, cell cycle progression and development. It has also been proven that MBD1 is involved in tumor development and progression. However, whether MBD1 is involved in tumorigenesis, especially in gallbladder cancer, is totally unknown.

**Methods:**

Human GBC-SD and SGC996 cells were used to perform experiments. Invasion, wound healing and colony formation assays were performed to evaluate cell viability. A CCK-8 assay was performed to assess gallbladder cancer cell viability after gemcitabine treatment. Western blot analysis was used to evaluate changes in protein expression. Human gallbladder cancer tissues and adjacent nontumor tissues were subjected to immunohistochemical staining to detect protein expression.

**Results:**

We found that MBD1 expression was significantly upregulated in gallbladder cancer tissues compared with that in surrounding normal tissues according to immunohistochemical analysis of 84 surgically resected gallbladder cancer specimens. These data also indicated that higher MBD1 expression was correlated with lymph node metastasis and poor survival in gallbladder cancer patients. Overexpression and deletion in vitro validated MBD1 as a potent oncogene promoting malignant behaviors in gallbladder cancer cells, including invasion, proliferation and migration, as well as epithelial–mesenchymal transition. Studies have demonstrated that epithelial–mesenchymal transition is common in gallbladder cancer, and it is well known that drug resistance and epithelial–mesenchymal transition are very closely correlated. Herein, our data show that targeting MBD1 restored gallbladder cancer cell sensitivity to gemcitabine chemotherapy.

**Conclusions:**

Taken together, the results of our study revealed a novel function of MBD1 in gallbladder cancer tumor development and progression through participation in the gallbladder cancer epithelial–mesenchymal transition program, which is involved in resistance to gemcitabine chemotherapy. Thus, MBD1 may be a potential therapeutic target for gallbladder cancer.

## Background

Gallbladder cancer (GBC) has a high diagnostic rate in Asia and southern America and is the most common and aggressive biliary tract cancer (BTC) [1–3]. GBC is characterized by local invasion, extensive regional lymph node metastasis, vascular encasement, and distant metastases [2]. Currently, complete surgical resection of the gallbladder offers the only opportunity for cure; however, only 10% of patients with GBC are considered surgical candidates [4]. Among those patients who do undergo “curative” resection, recurrence rates are high. The 5-year survival rate is still less than 5% due to late diagnosis, the low surgical resection rate, and the high recurrence rate coupled with metastatic features [1, 2, 4, 5].

For patients with unresectable advanced or metastatic GBC, chemotherapies are the main therapeutic regimens. Gemcitabine (Gem) is an effective chemotherapeutic agent for GBC [4, 6]. The tumor response rate to Gem was reported to vary between 10 and 30%, and the median survival time was 8.1 months [7, 8], which indicates that GBC is highly resistant to Gem, further increasing the challenge of GBC treatment [4]. Studies have demonstrated that epithelial–mesenchymal transition (EMT) is common in gallbladder cancer [9], breast cancer [10], colon cancer [11], ovarian cancer [12], and in a fraction of bladder cancer patients [13]. EMT increases the resistance of tumor cells to chemotherapeutic drugs when cells are transfected with some hallmark EMT genes, including Notch, Twist, and TGFβ [14, 15].

Methyl-CpG binding domain protein 1 (MBD1), which couples DNA methylation to transcriptional repression, has been implicated in transcriptional regulation, heterochromatin formation, genomic stability, cell cycle progression and development [16, 17]. It has also been shown that MBD1 is involved in tumor development and progression [17–20]. However, whether and how MBD1 is involved in GBC tumorigenesis and chemotherapeutic resistance are currently unknown. Research has revealed that epigenetic modifications, especially promoter hypermethylation, plays an important role in the 5-FU drug resistance of BTCs [21]. Miyazaki K found that expression of dihydropyrimidine dehydrogenase, a well-known key factor in 5-FU drug resistance, was suppressed by promoter hypermethylation [21]. This finding indicates that epigenetic methylation is closely related with drug resistance in BTCs. Herein, we conducted a study to investigate the role of MBD1 in GBC development and progression. Moreover, we evaluated whether MBD1 was involved in Gem resistance in GBC.

## Methods

### Patients, specimens, and cell lines

With approval by the ethics committee of Fudan University Shanghai Cancer Center (FUSCC), specimens of 84 GBC tissues and 57 adjacent nontumor tissues were obtained from patients who underwent surgery for GBC between January 2012 and December 2017 in FUSCC. Clinical information, including age, sex, TNM stage, pathological type, metastasis, neoplasm histological grade, tumor size, and months of follow-up, was collected.

The gallbladder cancer cell lines GBC-SD and SGC-996 were purchased from Shanghai Cell Bank (Shanghai, China) and incubated in a CO_2_ incubator (5% CO_2_/95% air) at 37 °C in DMEM supplemented with 10% fetal bovine serum.

### Immunohistochemical staining

Paraffin-embedded tissue slides were deparaffinized in xylene, rehydrated through a graded series of alcohol solutions, blocked in methanol containing 3% hydrogen peroxide, and incubated with anti-MBD1 antibody. Following rinsing with phosphate‑buffered saline (PBS) solution, slides were incubated with horseradish peroxidase-conjugated secondary antibodies at room temperature. Finally, slides were incubated with 3,3′-diaminobenzidine solution at room temperature for 10 min and counterstained with hematoxylin. Two experienced pathologists who were blinded to the clinicopathological data independently evaluated the immunostaining. The MBD1 staining patterns in sections were scored as follows: 0, no staining; 1+, weak staining; 2+, moderate staining; or 3+, strong staining. In addition, scores of 2+ and 3+ were defined as high expression, and the other scores were defined as low expression for statistical analysis.

### Western blotting

Western blotting was carried out as previously described [22]. Briefly, whole-cell protein lysates were extracted, separated by SDS-PAGE and subjected to immunoblotting. The antibodies used were purchased from Abcam (Cambridge, MA, USA).

### Lentivirus production and infection

GBC-SD and SGC-996 cell lines that stably expressed MBD1 and shRNA oligos directed against MBD1 were established by lentiviral-mediated transfection. The lentiviral vector pLKO.1-TRC (Addgene plasmid 10878) was used according to an online protocol (http://www.addgene.org/tools/protocols/plko/). Briefly, shRNA oligos targeting human MBD1 were designed and cloned into the pLKO.1-TRC cloning vector digested with EcoRI and AgeI. The recombinant construct, together with two packaging vectors, psPAX2 and pMD2.G, was transiently transfected into 293T cells. pLKO.1-scramble (SCR) shRNA (Addgene plasmid 1864) was used as the negative control. Lentiviral particles were harvested, filtered and used to infect target GBC cells. To overexpress MBD1, FLAG-tagged MBD1 was cloned into the lentiviral vector pWPI.1. Lentiviral particles were produced by cotransfection of pWPI.1-MBD1-FLAG with psPAX2 and pMD.G into 293T cells.

### In vitro Invasion assay and colony formation assay

The invasion assay was performed as previously described [23] using Transwell cell culture chambers (8 mM pore size polycarbonate membranes, Costar). Cells that invaded the membrane were counted in 10 randomly selected microscopic fields. Each assay was performed in triplicate. A colony formation assay was performed by seeding cells in triplicate in 6-well plates at an initial density of 500 cells per well. After 10–14 days, colonies were clearly visible, and cells were fixed with 4% paraformaldehyde for 15 min at room temperature and stained with 4 mg/ml crystal violet. Colonies containing more than 50 cells were counted using light microscopy. The average number of colonies was determined from three independent experiments.

### Cell viability assays

Cell viability was measured using a Cell Counting Kit-8 (Dojindo, Tokyo, Japan). Briefly, 200 μl of medium containing cells (3000 cells/well) was seeded in 96-well plates. After culturing for the indicated times, CCK-8 solution was added to each well at 37 °C. After 2 h, the optical density (OD) values of each well at a wavelength of 450 nm were measured using a microplate reader.

### Wound healing assay

An in vitro wounding assay was performed by creating a scratch with a 100 μl pipette tip on the surface of a confluent dish of GBC-SD, GBC-SD-MBD1, SGC-996 and SGC-996-MBD1 cells. Images were acquired and compared between the time of wounding and regular intervals during cell migration for wound closure.

### Statistical analysis

Experiments were repeated at least three times. All data are presented as the mean ± SD. Two-tailed unpaired Student’s t tests and one-way analysis of variance were used to evaluate the data. SPSS version 16.0 software (IBM) was used for data analysis. Differences were considered significant at *P *< 0.05.

## Results

### MBD1 expression level correlated with survival in GBC patients

**Fig. 1:**
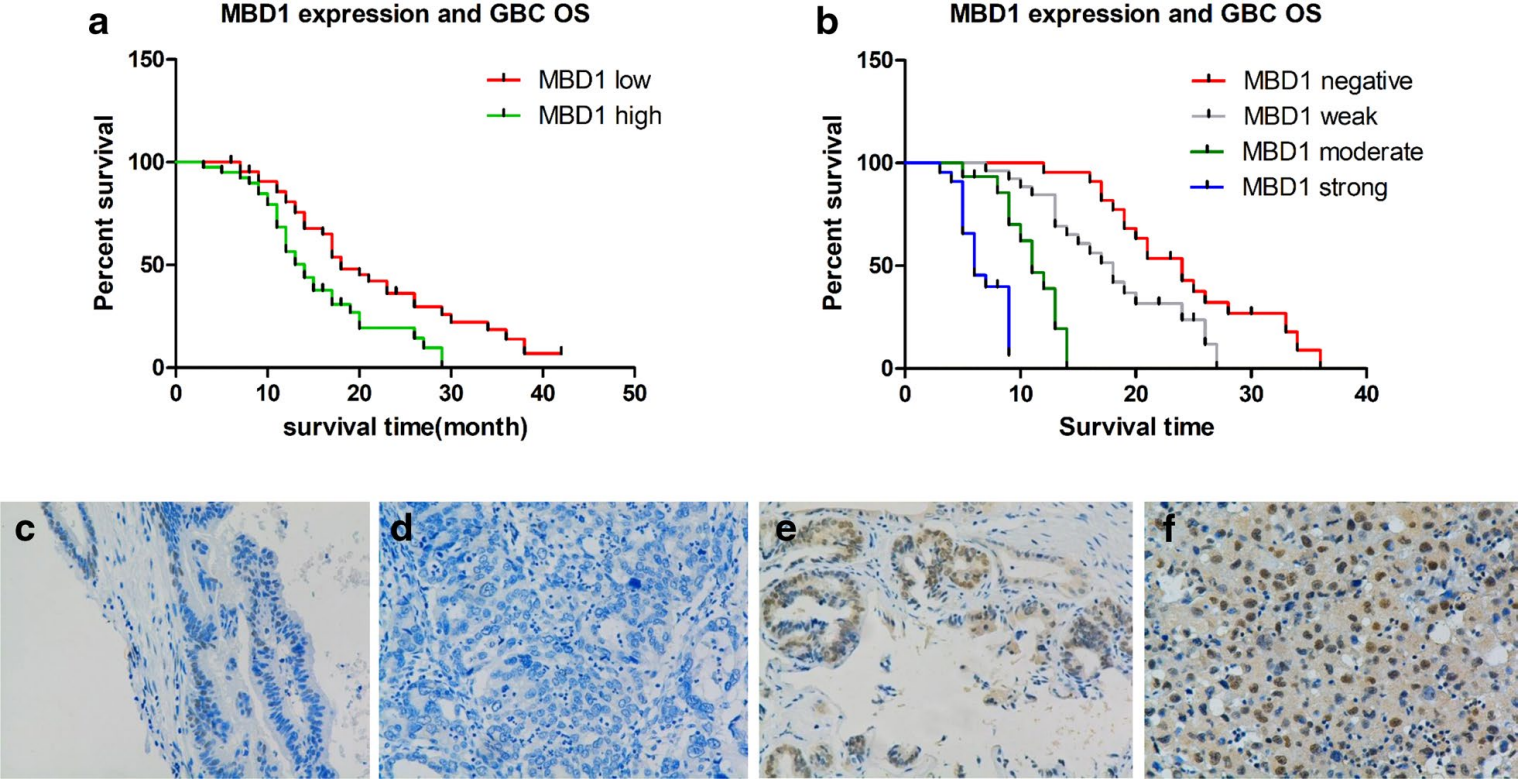
MBD1 expression is significantly related to the overall survival of patients with gallbladder cancer. **a** Survival analysis of MBD1 in the FUSCC dataset. High MBD1 expression indicated worse prognosis. *P *< 0.05 vs. the low group. **b** Survival analysis of patients with the indicated expression levels of MBD1 in the FUSCC dataset. *P *< 0.05 vs. the negative group. **c**–**f** IHC scoring of MBD1 expression in tissue samples from patients with GBC. **c** Negative; **d** low expression; **e** moderate expression; **f** high expression

To explore the impact of MBD1 on GBC patient prognosis, we examined MBD1 expression in 84 immunohistochemically stained GBC tissues from patients at our center between 2012 and 2017. First, we performed a detailed evaluation of MBD1 staining based on IHC scoring, and the scoring standard is shown in Fig. 1a.

We performed Kaplan–Meier analysis on patients stratified by MBD1 expression and further analyzed the MBD1 expression status and prognosis of patients with GBC from our center. These results demonstrated that the MBD1 expression level was significantly related to the overall survival (OS) of GBC patients (Fig. 1b). Moreover, the OS time of GBC patients with high MBD1 expression was significantly shorter than that of patients with low MBD1 expression (*P* < 0.001, median survival time: 24 months, 18 months, 11 months, and 6 months for IHC scores of 0, 1+, 2+, and 3+, respectively). Moreover, we analyzed the correlation between MBD1 expression and clinicopathological features in these 84 GBC samples. The results showed that high MBD1 expression was closely related to the lymph node metastasis status (Table 1, *P *< 0.001), distant metastasis status (Table 1, *P *= 0.006) and TNM stage (Table 1, *P *= 0.008). Thus, we hypothesized that MBD1 may play an important role in the development of GBC and could be closely related to prognosis in GBC.

**Table 1:** Relationship between MBD1 expression and clinicopathological factors of patients with GBC

Parameter	No. of patients	MBD1 (low)	MBD1 (high)	Spearman correlation	*P* value
Sex				0.107	0.333
Male	45	16 (35.6%)	29 (64.4%)		
Female	39	10 (25.6%)	29 (74.4%)		
Age (years)				− 0.020	0.859
< 60	40	12 (30.0%)	28 (70.0%)		
≥ 60	44	14 (31.9%)	30 (68.1%)		
Tumor size (cm)				0.085	0.441
≤ 5	53	18 (33.9%)	35 (66.1%)		
> 5	31	8 (25.8%)	23 (74.2%)		
Differentiation grade				0.182	0.098
Well-moderate	31	13 (41.9%)	18 (58.1%)		
Poor-undifferentiated	53	13 (24.5%)	40 (75.5%)		
T stage				0.036	0.742
T1–T3	56	18 (32.1%)	38 (67.9%)		
T4	28	8 (28.6%)	20 (71.4%)		
Lymph node status				0.378	< 0.001
Negative	55	24 (43.6%)	31 (56.4%)		
Positive	29	2 (6.9%)	27 (93.1%)		
Distant metastasis status				0.299	0.006
M0	53	22 (41.5%)	31 (58.5%)		
M1	31	4 (12.9%)	27 (87.1%)		
TNM stage				0.287	0.008
I–II	34	16 (47.1%)	18 (52.9%)		
III–IV	50	10 (20.0%)	40 (80.0%)		

MBD1 Low: negative/weak MBD1 expression; MBD1 High: moderate/strong MBD1 expression; T stage and TNM stage were defined by the AJCC 8th edition; *P*-values were derived by Spearman rank correlation; all statistical tests were two-sided

### MBD1 expression affects GBC cell proliferation, invasion and migration in vitro

**Fig. 2:**
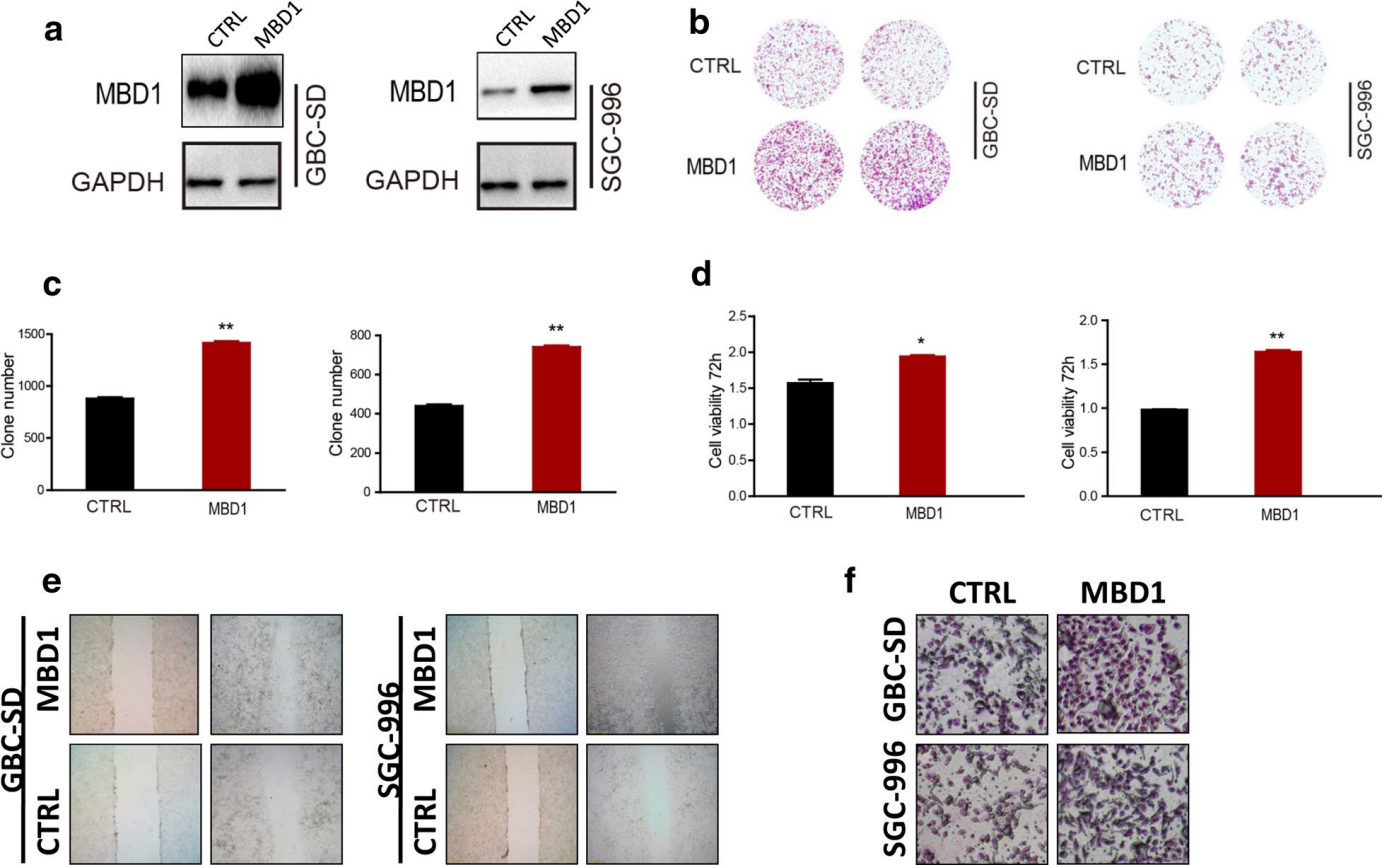
MBD1 enhances the proliferation, invasion and migration capabilities of GBC cells in vitro. **a** MBD1-overexpressing cell clones were generated with GBC-SD and SGC-996 cells. **b**, **c** Overexpression of MBD1 significantly increased the colony-forming capacity of GBC-SD and SGC-996 cells. **d** A CCK-8 proliferation assay showed that MBD1 overexpression significantly elevated the viability of GBC-SD and SGC-996 cells. **e, f** Wound healing and Transwell assays showed that MBD1 promoted the invasion and migration capabilities of GBC cells. *P < 0.05, **P < 0.01

To further evaluate the function of MBD1 in GBC viability and proliferation, we generated an MDB1 expression vector to induce MBD1 overexpression in GBC-SD and SGC-996 cells. The efficiency of overexpression was validated by western blotting (Fig. 2a).

Then, we performed a colony formation assay. These results revealed that overexpression of MBD1 significantly increased the colony formation capacity of GBC-SD and SGC-996 cells, supporting a role for MBD1 in GBC cell proliferation (Fig. 2b, c). Moreover, we also performed CCK-8 proliferation assays to validate the influence of MBD1 on GBC cell viability. As shown, MBD1 overexpression significantly elevated the viability of GBC-SD and SGC-996 cells (Fig. 2d). The effect of MBD1 on invasion and migration was also investigated by a wound healing assay and Transwell assay in the two GBC cell lines, which further confirmed that MBD1 promoted the invasion and migration capabilities of GBC cells (Fig. 2e, f).

**Fig. 3:**
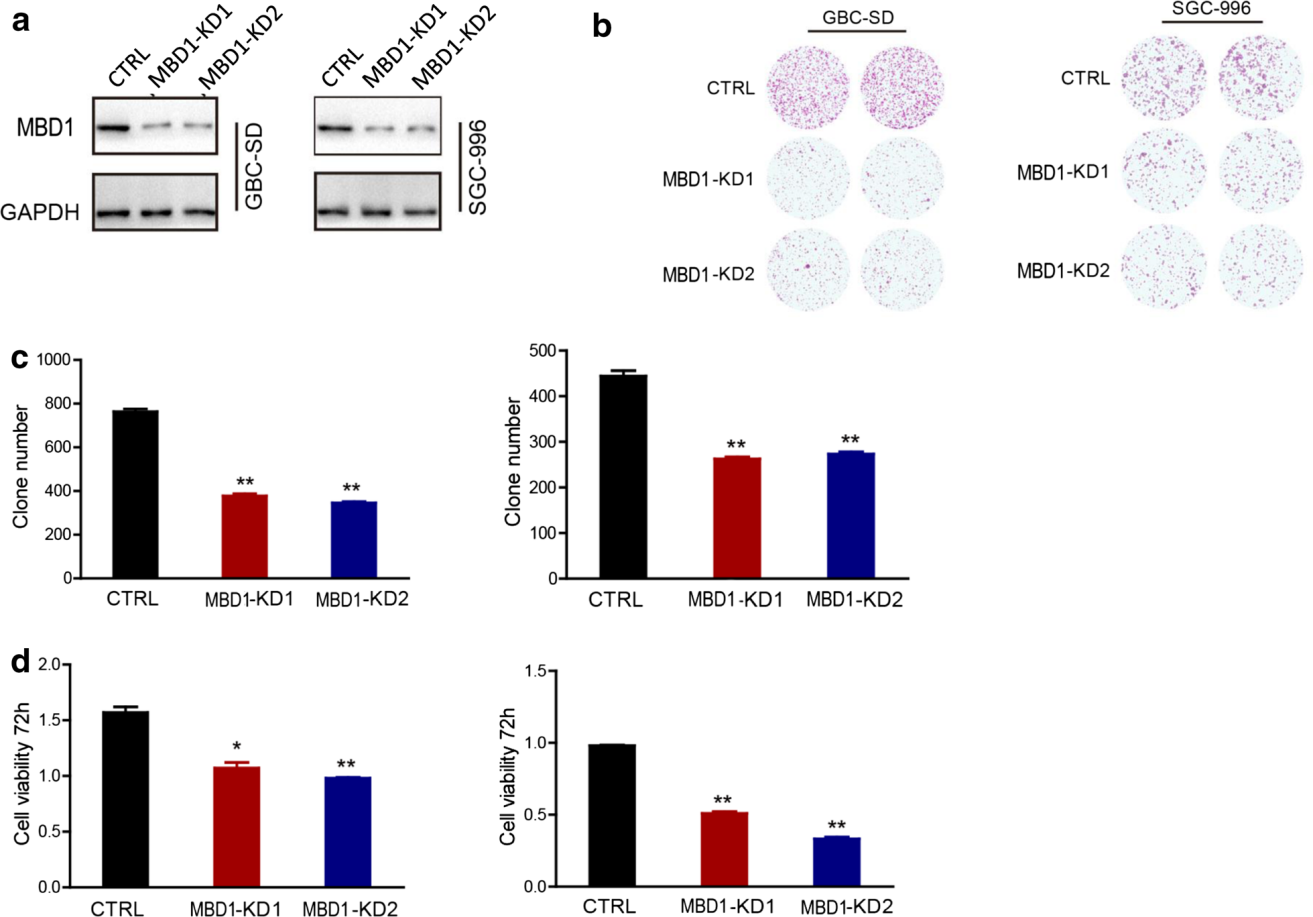
Silencing MBD1 expression inhibited GBC cell viability and proliferation. **a** MBD1 knockdown cell clones were generated with GBC-SD and SGC-996 cells. **b**–**d** Colony formation and CCK-8 proliferation assays confirmed that silencing MBD1 expression significantly reduced the viability of GBC-SD and SGC-996 cells relative to that of control cells. *P < 0.05, **P < 0.01

To further prove that the observed enhancement of proliferation, invasion and migration was not due to mixed factors, we constructed lentiviral particles targeting MBD1, termed MBD1 KD1 and MBD1 KD1, to silence MBD1 expression. The knockdown efficiency was validated by western blotting, as before (Fig. 3a). Again, colony formation assays and CCK-8 proliferation assays were performed to observe the effect of MBD1 on GBC cell viability and proliferation. As expected, MBD1 knockdown significantly reduced the viability of GBC-SD and SGC-996 cells (Fig. 3b–d).

### MBD1 induces EMT in GBC cancer cells

**Fig. 4:**
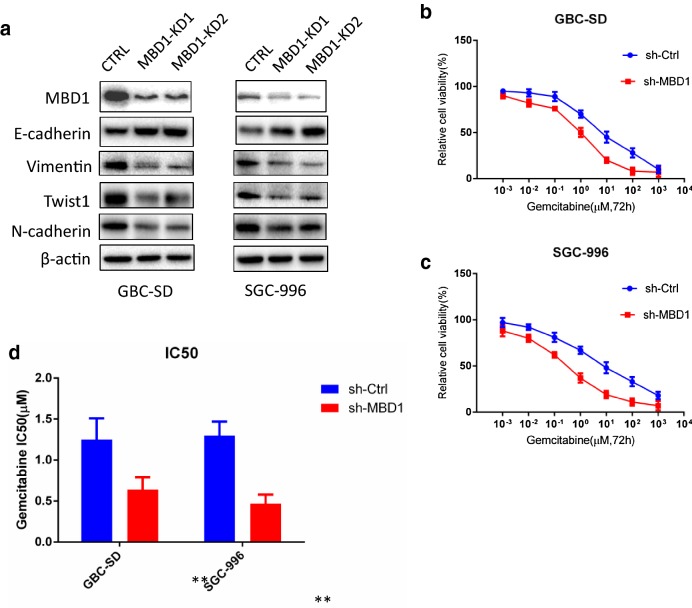
MBD1 induces EMT in GBC cancer cells and plays a role in the chemosensitivity of GBC cells. **a** MBD1 silencing resulted in increased expression of the epithelial marker E-cadherin and inhibition of mesenchymal markers, including Twist1, N-cadherin and Vimentin. **b**–**d** GBC cell chemosensitivity to Gem was evaluated in MBD1 knockdown cell clones. **b** The Gem sensitivity of GBC-SD cells with downregulation of MBD1 expression was significantly enhanced relative to that of control cells. **c** MBD1 knockdown elevated the Gem sensitivity of SGC-996 cells relative to that of control cells. **d** Inhibition of MBD1 expression dramatically decreased the IC50 of Gem in GBC-SD and SGC-996 cells. *P < 0.05, **P < 0.01

To better understand the regulatory mechanisms of MBD1 in GBC progression, we investigated the expression of EMT-related proteins by western blotting in established MBD1 knockdown GBC cell lines. As shown in Fig. 4a, when the MBD level was decreased, the expression of the epithelial marker E-cadherin increased, indicating that MBD1 may suppress the expression of E-cadherin and promote EMT in GBC cells. Furthermore, MBD1 knockdown by shRNA in GBC cells induced the inhibition of mesenchymal markers, including Twist1, N-cadherin and Vimentin (Fig. 4a). Given these data, we hypothesized that MBD1 down-regulates E-cadherin expression while upregulating the expression of mesenchymal-related proteins in GBC, resulting in a shift in the EMT phenotype in GBC cells. This phenotypic shift may play an important role in GBC invasion and metastasis.

### MBD1 modulates the chemosensitivity of GBC cells

We hypothesized that MBD1 is involved in the chemosensitivity of GBC and might be a novel target for clinical intervention. We examined chemosensitivity to Gem in well-established MBD1 knockdown GBC cells. With downregulation of MBD1 expression, the Gem sensitivity of GBC-SD cells was significantly enhanced compared with that of control cells (Fig. 4b). Similarly, MBD1 knockdown also elevated the Gem sensitivity of SGC-996 cells compared with that of control cells (Fig. 4c). We further compared the IC50 of Gem in both GBC-SD and SGC-996 cells. As shown in Fig. 4d, inhibition of MBD1 expression dramatically decreased the IC50 of Gem in GBC-SD and SGC-996 cells. Collectively, these results indicated that MBD1 may strongly affect Gem chemosensitivity in GBC cells. We propose that this effect was mediated by the phenotypic shift toward EMT.

## Discussion

**Fig. 5:**
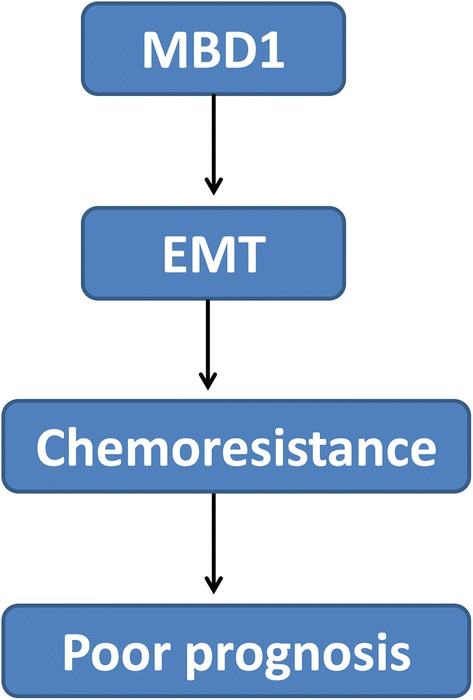
Schematic summarizing the role of MBD1 in regulating the malignant behavior of GBC cells, as indicated by this investigation

Previous studies have demonstrated that MBD1 may contribute to tumorigenesis by binding to hypermethylated CpG islands in the promoters of tumor suppressor genes in cancer cells, for example, in pancreatic cancer [24], lung cancer [19], prostate cancer [25] and leukemia [26] cells. However, whether MBD1 is also involved in regulating GBC tumorigenesis, especially in terms of chemoresistance, has never been investigated. In the present study, we found that the MBD1 expression level is correlated with GBC patient prognosis (Fig. 5). Reducing MBD1 expression inhibited the proliferation, invasion and migration capabilities of GBC cells, while enhancing MBD1 expression augmented these malignant behaviors (Fig. 5). Further investigation revealed that MBD1 is involved in Gem chemoresistance through EMT modulation in GBC cells (Fig. 5).

MBD1, the largest member of the MBD family [27], plays a major role in coordinating crosstalk between DNA methylation, histone modification and chromatin organization to achieve a coherent transcriptional program [16, 28]. MBD proteins have been implicated in many kinds of human cancers, but the precise roles of MBD proteins differ between types of cancer [16, 27–29]. Regarding MBD1, previous reports have revealed its double–faced role in tumorigenesis [19, 30]. Considering the complicated role of MBD1 in different cancers, herein, we first investigated its expression in GBC tissues and explored the correlation between its expression level and clinical prognosis (Table 1). We found that a higher level of MBD1 expression indicated a worse prognosis, and further analysis based on IHC scoring demonstrated that the MBD1 level was strongly related to the OS time of GBC patients (Table 1). Moreover, MBD1 expression levels were significantly correlated with tumor differentiation and lymph node metastasis status (Table 1). Our data provide evidence that MBD1 could be a vital player in promoting the progression of GBC. Interestingly, previous studies by our team also found high expression of MBD1 in pancreatic cancer cell lines and tissues [20, 23, 24, 31]. When MBD1 expression was knocked down in vitro, pancreatic cancer cell growth was inhibited and apoptosis was induced. Indeed, in the present study, we enhanced the expression of MBD1 in GBC cancer cell lines and evaluated the proliferation and migration capabilities (Fig. 2). Unsurprisingly, MBD1 overexpression dramatically promoted the proliferation and migration capabilities of GBC cells (Fig. 2). To further validate the above results, MBD1 was knocked down in the two indicated GBC cell lines. As expected, decreased MBD1 expression resulted in diminished cell proliferation and viability (Fig. 3). Collectively, these data proved that MBD1 is an important molecule in mediating the malignant behavior of GBC cells.

Generally, only a few GBC patients are diagnosed with early-stage disease and can be treated with curative surgery. However, for patients diagnosed with advanced disease, treatment options are noncurative and mainly chemotherapy-based [4]. Data from randomized trials have demonstrated that systemic chemotherapy prolongs the survival and improves the quality of life in patients with metastatic BTC [32]. BTC, including cholangiocarcinoma (both intra- and extrahepatic) and GBC, have common features, such as a highly desmoplastic reaction, a rich tumor microenvironment, and profound genetic heterogeneity; all of these features contribute to the development of drug resistance and the almost complete absence of curative therapies for metastatic disease [33]. Most completed trials have been and most ongoing trials are being conducted in BTC. Thus, we generally discuss the treatment of GBC in the context of BTC. The first study to suggest that palliative chemotherapy could improve survival and quality of life was reported in 1996 [32]. Gem had been established as a standard treatment option for patients with hepatobiliary tumors [34]. As a single agent, Gem has shown a response rate ranging from 0 to 30%, indicating a high probability of resistance to Gem chemotherapy in GBC [8]. Indeed, chemoresistance has long been an unsolved issue implicated in GBC patients’ poor prognosis [4, 35, 36]. The identification of novel regulators involved in chemotherapeutic inefficiency is urgently needed to overcome this obstacle.

EMT has a prominent role in the early steps of tumor progression and metastasis, as well as in drug resistance [14, 15, 37]. Accumulating evidence suggests that EMT could be critical in regulating tumor progression and poor prognosis in GBC [9, 38, 39]. In the present study, we attenuated MBD1 expression in GBC cell lines and found that E-cadherin expression was strikingly upregulated (Fig. 4). The results also showed that the expression of the mesenchymal markers Vimentin and N-cadherin was decreased dramatically in association with MBD1 deletion (Fig. 4). Our investigation indicated that MBD1 modulated EMT to participate in tumor progression, implying connections between MBD1 and resistance to Gem chemotherapy in GBC. To verify this possibility, we examined the chemosensitivity of GBC cancer cells to Gem. Gem sensitivity was significantly elevated in GBC cell lines with deletion of MBD1 (Fig. 4). Moreover, inhibition of MBD1 expression dramatically decreased the IC50 of Gem (Fig. 4). In the present study, we established the role of MBD1 in GBC progression and chemoresistance.

In addition, combining Gem with other candidate drugs is another strategy to improve outcomes. Following the results of Valle’s phase 3 ABC-02 trial (median PFS of 8.0 months and median OS of 11.7 months) cisplatin/Gem has become a recognized standard regimen for the first-line treatment of patients with advanced BTC [8]. Recently, the introduction of nab-paclitaxel has shed light on treatment options for chemotherapy in BTC. Paclitaxel can inhibit the Gem-metabolizing enzyme cytidine deaminase to increase the intratumoral concentration of active Gem metabolites. However, standard paclitaxel has considerable toxicity compared with the nanoparticle albumin-bound (nab) colloidal formulation, nab-paclitaxel (nabP), which is associated with a lower incidence of vehicle-related hypersensitivity reactions, neurotoxicity, and neutropenia [40]. In a phase 2 clinical trial, Gem plus nabP was used as a first-line treatment for advanced or metastatic cholangiocarcinoma (GBC excluded) [41]. The PFS rate at 6 months was observed to be 61% in the intention-to-treat population. The primary endpoint in this trial, along with the secondary efficacy endpoints of a median PFS time of 7.7 months and a median OS time of 12.4 months, were similar to that in the phase 3 ABC-02 trial (median PFS time of 8.0 months and median OS time of 11.7 months) [8, 41]. These results indicate that a nab-paclitaxel plus Gem regimen was well tolerated and may be an alternative option to current therapeutic approaches for advanced cholangiocarcinoma. In another phase 2 clinical trial [42], administration of nabP, Gem and cisplatin resulted in a median progression-free survival time of 11.8 months and a median overall survival time of 19.2 months in an intention-to-treat analysis. The partial response rate was 45%, and the disease control rate was 84%. Administration of nab-paclitaxel plus Gem-cisplatin may result in longer survival than administration of Gem-cisplatin alone in patients with advanced BTC. These findings still need to be tested further in a phase 3 randomized clinical trial.

After the failure of first-line therapy, approximately half of the patients still have a good performance status and satisfactory organ function [43], but the advantages of second-line therapy are still unclear, and no quality evidence is available to support the use of second-line chemotherapy [44]. To date, the role of second-line therapy is unclear; no single regimen has emerged. The most common regimens used are 5-FU/folinic acid, FOLFIRI, XELIRI, FOLFOX, XELOX, 5-FU and cisplatin; however, the outcomes are generally poor [45]. Ongoing trials are trying to address this lack of treatment options, highlighting the need for the development of novel targeted therapy approaches. In the past decade, we have entered the era of targeted therapies. Currently, the most promising targets under development, due to their relatively solid preclinical research background, are IDH inhibitors for IDH-mutant BTC and molecules targeting FGFR2 gene fusions [46]. Most of the remaining molecular targets that have been tested in clinical trials have been somewhat disappointing, with conflicting data and negative trials [46]. Thus, new models and new approaches to unravel the complex molecular biology of BTC are needed. Here, we identify MBD1 as another potential molecule that could be a promising target for the development of new treatment options for BTCs, especially for GBC.

## Conclusion

In summary, this study is the first to reveal the important role of MBD1 in modulating the malignant behavior and poor prognosis of GBC. MBD1 affects the chemosensitivity of GBC to Gem and potentially achieves this effect by mediating the EMT program. This observation provides clues and new insight into the development of new therapeutic targets to overcome obstacles in GBC treatment. Taken together, our results indicate that MBD1 is a valuable prognostic marker and an important treatment target for GBC. Further understanding of the molecular mechanism of MBD1 in mediating EMT and drug resistance in GBC will help to assess the therapeutic relevance of targeting a specific pathway.

## Data Availability

All data generated or analyzed during this study are included in this published article.

## Abbreviations

MBD1: methyl-CpG binding domain protein 1
GBC: gallbladder cancer
EMT: epithelial–mesenchymal transition
Gem: gemcitabine
BTCs: biliary tract cancers
PBS: phosphate‑buffered saline
CCK-8: cell counting kit-8
OS: overall survival
nabP: nab-paclitaxel
